# Editorial: Antibody-mediated rejection

**DOI:** 10.3389/frtra.2024.1408225

**Published:** 2024-04-10

**Authors:** Ramsey R. Hachem, Thalachallour Mohanakumar

**Affiliations:** ^1^ Division of Pulmonary and Critical Care, Washington University in St. Louis, St. Louis, MO, United States; ^2^ St. Joseph’s Hospital and Medical Center, Norton Thoracic Institute, Phoenix, AZ, United States

**Keywords:** antibody-mediated rejection, editorial, immunosuppression, solid organ transplantation, graft failure

**Editorial on the Research Topic** Antibody-mediated rejection

Current therapeutic immunosuppression, focused on inhibiting *T* cell activation and proliferation, has made solid organ transplantation a clinically feasible treatment for advanced diseases and organ failure. Indeed, transplantation has been a life-saving therapy for nearly 1 million individuals in the United States alone since 1988 (1). However, long-term outcomes remain disappointing, especially after lung transplantation, and antibody-mediated rejection (AMR) has emerged as a significant barrier to better graft and patient survival after all solid organ transplants. This Research Topic highlights recent advances and persistent challenges in AMR after solid organ transplantation.

Dr. Franco-Acevedo et al. review recent advances in our understanding of mechanisms that lead to AMR, focusing on transplant vasculopathy. The authors highlight how antibodies bind to mismatched human leukocyte antigens (HLA) on endothelial cells to promote proinflammatory signaling and the expression of adhesion molecules and chemokines. During AMR, this can result in endothelial to mesenchymal transition which can lead to unresolved chronic repair responses and maladaptive vascular remodeling. In the kidney, this is recognized as transplant vasculopathy which causes narrowing of the vascular lumen and ultimately graft failure. The authors note that there are organ specific differences in endothelial responses to antibody binding, and these have not been fully elucidated. Dr. Bansal et al. outline the pathogenesis of AMR as B cells or plasma cells producing antibodies against HLA that interact with endothelial cells to activate signaling pathways and recruit immune cells. The authors further note that AMR may be complement-dependent or complement-independent. Furthermore, extracellular vesicles (EV) have been evaluated as potential biomarkers in transplantation. EVs are released from different cell types and are present in body fluids including blood, urine, and bronchoalveolar lavage (BAL) fluid. The composition and surface markers of EVs vary depending on the cell of origin. In lung transplantation, EVs and their contents can serve as a non-invasive biomarker for the development of chronic lung allograft dysfunction (CLAD) and can distinguish between the two phenotypes of CLAD: bronchiolitis obliterans syndrome and restrictive allograft syndrome (2, 3).

Dr. Yang et al. summarize the latest findings defining autoimmune responses after lung transplantation. The prototypic self-antigens include collagen V and K-α 1 tubulin but other less well investigated self-antigens are angiotensin II type 1 receptor (AT1R) and endothelin-1 type A receptor (ETAR) (4, 5). The pathogenesis of the development of autoimmune responses involves exposure of normally cryptic self-antigens following transplant-associated injury (e.g., ischemia-reperfusion injury, gastroesophageal reflux with aspiration) followed by loss of peripheral tolerance. It has been noted that respiratory viral infections can deplete regulatory *T* cells (Tregs) and thus promote the development of autoimmune responses to self-antigens. Antibodies to self-antigens have been identified as significant risk factors for primary graft dysfunction (PGD) after lung transplantation and CLAD (4). However, the optimal clinical approach to identifying these antibodies is yet to be defined.

Dr. Knechtle et al. highlight translating therapeutic strategies from non-human primate (NHP) models to clinical trials focused on desensitization before transplantation and AMR after kidney transplantation. Novel immune-suppressants and protocols consisting of multiple novel agents are best evaluated in randomized controlled trials. NHP models can be crucial to carefully design different immunosuppressive protocols. In this review, the authors highlight NHP work that led to two clinical trials examining desensitization regimens. NHP models identified the need to target multiple pathways to deplete antibodies and block further antibody production. Thus, two clinical trials are assessing the combinations of carfilzomib and belatacept and daratumumab and belatacept to deplete HLA antibodies and make kidney transplantation feasible. Finally, the authors review a planned clinical trial combining carfilzomib and belatacept for the treatment of AMR after kidney transplantation. Finally, Dr. Brandon et al. review the impact of donor-specific HLA antibodies (DSA) on outcomes after lung transplantation including the development of AMR and CLAD. The authors note that DSA are common after lung transplantation (6), and although their impact on outcomes has been well established, the optimal therapeutic approach after the development of DSA remains unknown.

AMR remains a significant problem in solid organ transplantation, and outcomes after AMR are poor. Allo-sensitization before transplantation is a barrier to transplantation and increases the risk of death on the waiting list (7). Importantly, desensitization remains difficult, and patients who are successfully desensitized and proceed to transplant are at increased risk of AMR and graft failure. Additional research is necessary to better understand the mechanisms that lead to AMR and how AMR results in graft failure. Better understanding of the underlying mechanisms will likely identify therapeutic targets. Ultimately, clinical trials will be necessary to critically examine the safety and efficacy of novel treatment regimens.
